# Research on bearing fault diagnosis based on improved genetic algorithm and BP neural network

**DOI:** 10.1038/s41598-024-66318-0

**Published:** 2024-07-05

**Authors:** Zenghua Chen, Lingjian Zhu, He Lu, Shichao Chen, Fenghua Zhu, Sheng Liu, Yunjun Han, Gang Xiong

**Affiliations:** 1 State Key Laboratory of Multimodal Artificial Intelligence Systems, Institute of Automation, Chinese Academy of Sciences, Beijing, 100190 China; 2 School of Mechanical and Precision Instrument Engineering, Xi’an University of Technology, Xi’an, 710048 China; 3 School of Education Renmin University of China, Beiing, 100872 China; 4 Beijing Engineering Research Center of Intelligent Systems and Technology, Institute of Automation, Chinese Academy of Sciences, Beijing, 100190 China; 5 Guangdong Engineering Research Center of 3D Printing and Intelligent Manufacturing, Cloud Computing Center, Chinese Academy of Sciences, Dongguan, 523808 China

**Keywords:** Rolling bearings, Fault diagnosis, Genetic algorithm, BP neural network, Optimization, Mechanical engineering, Computer science, Statistics, Actuators

## Abstract

Health monitoring and fault diagnosis of rolling bearings are crucial for the continuous and effective operation of mechanical equipment. In order to improve the accuracy of BP neural network in fault diagnosis of rolling bearings, a feature model is established from the vibration signals of rolling bearings, and an improved genetic algorithm is used to optimize the initial weights, biases, and hyperparameters of the BP neural network. This overcomes the shortcomings of BP neural network, such as being prone to local minima, slow convergence speed, and sample dependence. The improved genetic algorithm fully considers the degree of concentration and dispersion of population fitness in genetic algorithms, and adaptively adjusts the crossover and mutation probabilities of genetic algorithms in a non-linear manner. At the same time, in order to accelerate the optimization efficiency of the selection operator, the elite retention strategy is combined with the hierarchical proportional selection operation. Using the rolling bearing dataset from Case Western Reserve University in the United States as experimental data, the proposed algorithm was used for simulation and prediction. The experimental results show that compared with the other seven models, the proposed IGA-BPNN exhibit superior performance in both convergence speed and predictive performance.

## Introduction

Rolling bearings are one of the important components of rotating machinery, widely used in industrial fields such as production workshops, aerospace, aviation, industrial aircraft, and intelligent manufacturing. Rolling bearings are subjected to multiple mechanical stresses such as friction, vibration, and high-speed motion during high-speed rotation, which can lead to fatigue cracks and wear on their surfaces, re-sulting in structural failures and ultimately bearing failure. Failed bearings will lead to more energy consumption, while also subjecting the surrounding environment to ex-cessive heat and pressure, thereby shortening the overall lifespan of machinery and equipment. According to relevant data statistics, about 45% of rotating machinery equipment failures are caused by damage to rolling bearings, and about 40% of motor equipment failures are caused by rolling bearing failures^1,2^. With the development of modern industrial and manufacturing systems, the diagnosis of bearing faults and health monitoring of working conditions in rolling bearings are of great significance for ensuring the continuous and effective operation of related mechanical systems. The essence of fault diagnosis is to analyze the collected mechanical equipment signals and model information, in order to achieve effective diagnosis and accurate analysis of the working status of mechanical equipment^3^.

At present, scholars in many literatures obtain feature parameters through signal processing methods and combine them with recognition algorithms for fault recogni-tion, such as support vector machines^4,5^, extreme learning machines^6,7^, tradi-tional neural networks^8^, recurrent neural networks^9^, and convolutional neural networks^10,11^, etc.

Chen Xinyang et al. proposed a support vector machine algorithm (MFDE-SVM) based on multi-scale dilation and dispersion entropy to address the problem of difficult recognition of bearing fault signals in rotating machinery equipment. Multiscale di-lation and dispersion entropy (MFDE) captures the diversity and irregularity of data by analyzing data changes at different time scales, extracts fault features hidden in bearing vibration signals, and improves the accuracy of support vector machine (SVM) classification in bearing fault diagnosis^12^. Xinran et al.^13^ proposed a fault di-agnosis method for rolling bearings, which uses an improved sparrow search algorithm (SSA) to optimize support vector machine (SVM). The optimized SVM can achieve more accurate self-adaptive classification results. Abed et al.^14^ used discrete wavelet transform to extract fault features of rolling bearings, and then used recurrent neural networks for fault detection and classification. Janssens et al.^15^ proposed a convolutional neural network for autonomous learning of bearing fault features, which can achieve better results than traditional methods; He et al.^16^ proposed a bearing fault diagnosis model that combines autoencoder and discrete Fourier trans-form for bearing fault signal preprocessing, feature extraction, and fault classification. Ning et al.^17^ proposed an improved fish swarm algorithm that utilizes its global optimization ability to optimize the weights and thresholds of the BP neural network, forming a fault diagnosis method based on the improved fish swarm algorithm optimized neural network. Chenlin et al.^18^ proposed an improved CNN fault diagnosis method to address the uncertainty of manually selecting features in ex-isting rolling bearing fault diagnosis models and the problem that diagnostic models are not targeted. Lizhi et al.^19^ proposed an enhanced deep autoencoder net-work that combines the grey wolf algorithm to automatically select key network pa-rameters. This enhanced deep autoencoder network has better feature extraction abil-ity and stability. An et al.^20^ proposed a rolling bearing fault diagnosis algorithm based on overlapping group sparse model deep complex convolutional neural network to address the difficulty of feature extraction and multi-scale problems in composite signals of rolling bearings.

In most literature, optimization algorithms are used to optimize recognition net-works, but some networks do not reduce errors by adjusting weights and thresholds when approximating functions. For example, RBF networks reduce errors by adjusting Euclidean distances, while BP neural networks reduce approximation errors by ad-justing input weights and thresholds. Moreover, at the same accuracy, the structure of BP networks is more straightforward than other networks. In order to improve the recognition effect of rolling bearing faults, this paper uses an improved genetic algorithm to optimize the BP neural network^21–25^.

In order to improve the accuracy of BP neural network in bearing fault diagnosis, an improved genetic algorithm is used to optimize the initial weights, biases, and hy-perparameters of the BP neural network, in order to overcome the disadvantages of BP neural network being prone to local minima, slow convergence speed, and sample de-pendency. The improved genetic algorithm fully considers the degree of concentration and dispersion of population fitness in genetic algorithms, and adaptively adjusts the crossover and mutation probabilities of genetic algorithms in a non-linear manner, which avoids the problems of traditional genetic algorithm optimization neural network models easily falling into local optima and low solving efficiency. At the same time, in order to accelerate the optimization efficiency, a layered and propor-tional selection operator is adopted, which not only considers retaining elite individu-als but also improves the diversity of the population.

In order to verify the effectiveness of the IGA-BPNN algorithm proposed in this paper, the algorithm that only optimizes neural network weights proposed in this paper is defined as the IGA-BPNN-1 algorithm, and the algorithm that only optimizes neural network hyperparameters proposed in this paper is defined as the IGA-BPNN-2 algorithm. The algorithm that optimizes neural network weights and hyperparameters using traditional genetic algorithms is defined as GA-BPNN, the algorithm that only optimizes neural network weights using traditional genetic algorithms is defined as GA-BPNN-1 algorithm, and the algorithm that only optimizes neural network hyperparameters using traditional genetic algorithms is defined as GA-BPNN-2 algorithm. Simultaneously, an unoptimized BPNN algorithm is introduced as the baseline to compare the performance and efficiency of these seven algorithms. The above 7 methods all use the features extracted in this article for training and prediction. Then, in order to verify the effectiveness of the 23 feature models extracted in this paper, the method proposed in paper^27^ was also used to extract features, and the improved algorithm IGA-BPNN proposed in this paper was used for training and prediction, which was defined as IGA-BPN-27. This article conducted rich experiments using the eight algorithms mentioned above, and conducted detailed experiments and comparative analysis from the aspects of model error, algorithm convergence speed and computational efficiency, fault diagnosis performance, and prediction result volatility, proving the superiority of the proposed algorithm IGA-BPNN. Finally, the method proposed in paper^27^ was used to extract features through experiments using CNN and LSTM models. The experimental results showed that the IGA-BPNN method proposed in this paper still has significant advantages in computational efficiency and fault diagnosis performance.

## Selection of fault diagnosis characteristic indicators

This article uses the bearing dataset from Case Western Reserve University (CWRU) in the United States for experiments. The CWRU bearing fault dataset is a publicly available dataset commonly used for mechanical fault diagnosis and predic-tion research. It is provided by the Department of Mechanical Engineering at Case Western Reserve University and is used for the analysis and algorithm development of bearing fault diagnosis and prediction. This dataset contains four different types of bearing operating state data: inner ring failure, outer ring failure, rolling ball failure, and normal state. Each type of fault includes data from multiple operating conditions, such as different loads and speeds. The dataset collected vibration signals of bearings under different fault states, and sampled and processed them. Multiple bearing fault characteristic indicators can be extracted from the Case Western Reserve Universi-ty(CWRU) bearing fault dataset for fault diagnosis and prediction analysis. The fol-lowing are some common bearing fault characteristic indicators. (1) Vibration signal characteristics: including peak value, root mean square (RMS), pulse factor, etc. (2) Frequency domain characteristics: Peak frequency, Fault frequency of bearings. (3) Statistical characteristics: Mean, Variance, Skewness, Kurtosis, etc. (4) Wavelet packet features: Wavelet packet energy, etc.

**Figure 1:**
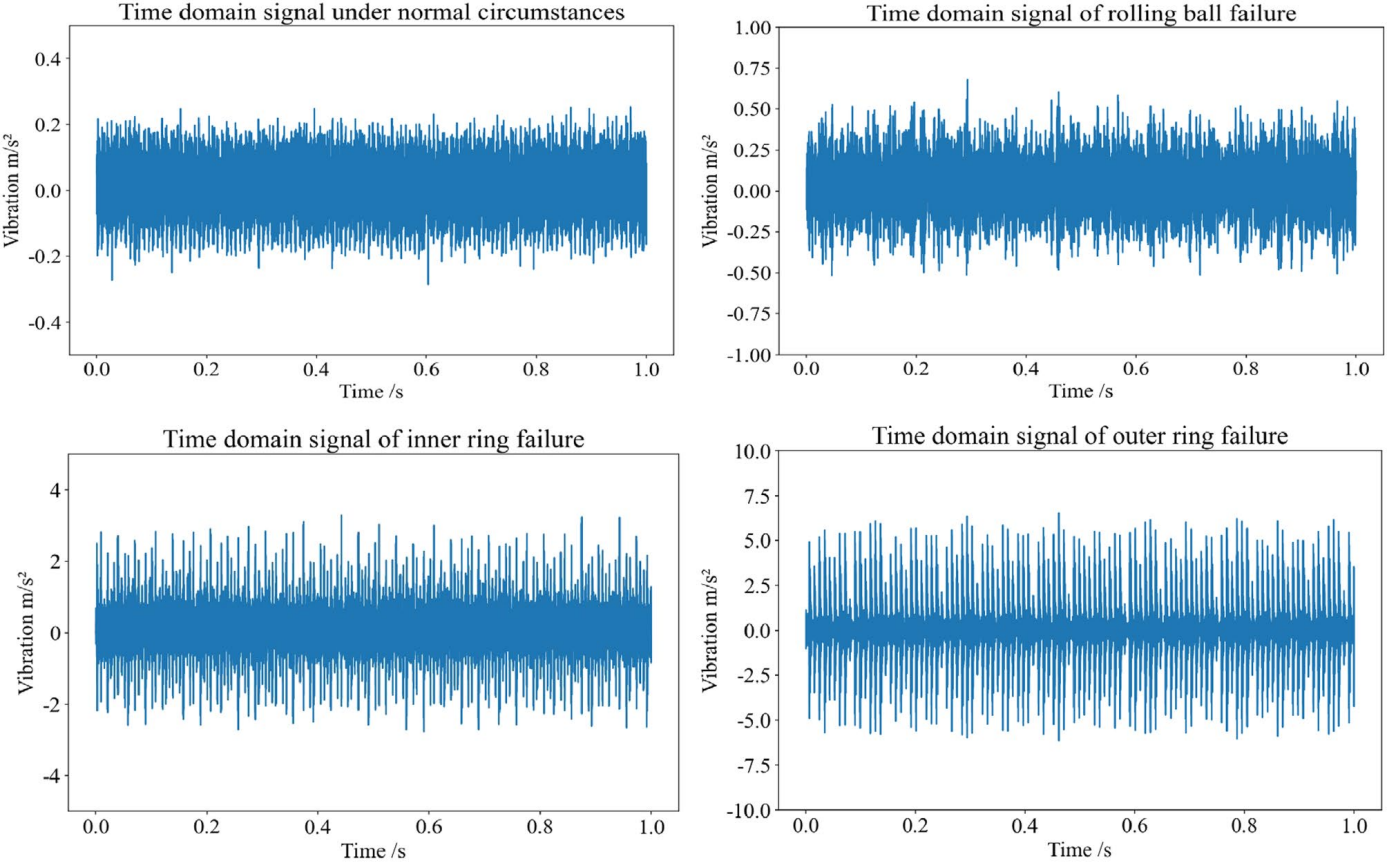
Time domain waveform of bearing vibration signal under 0hp working condition.

**Figure 2:**
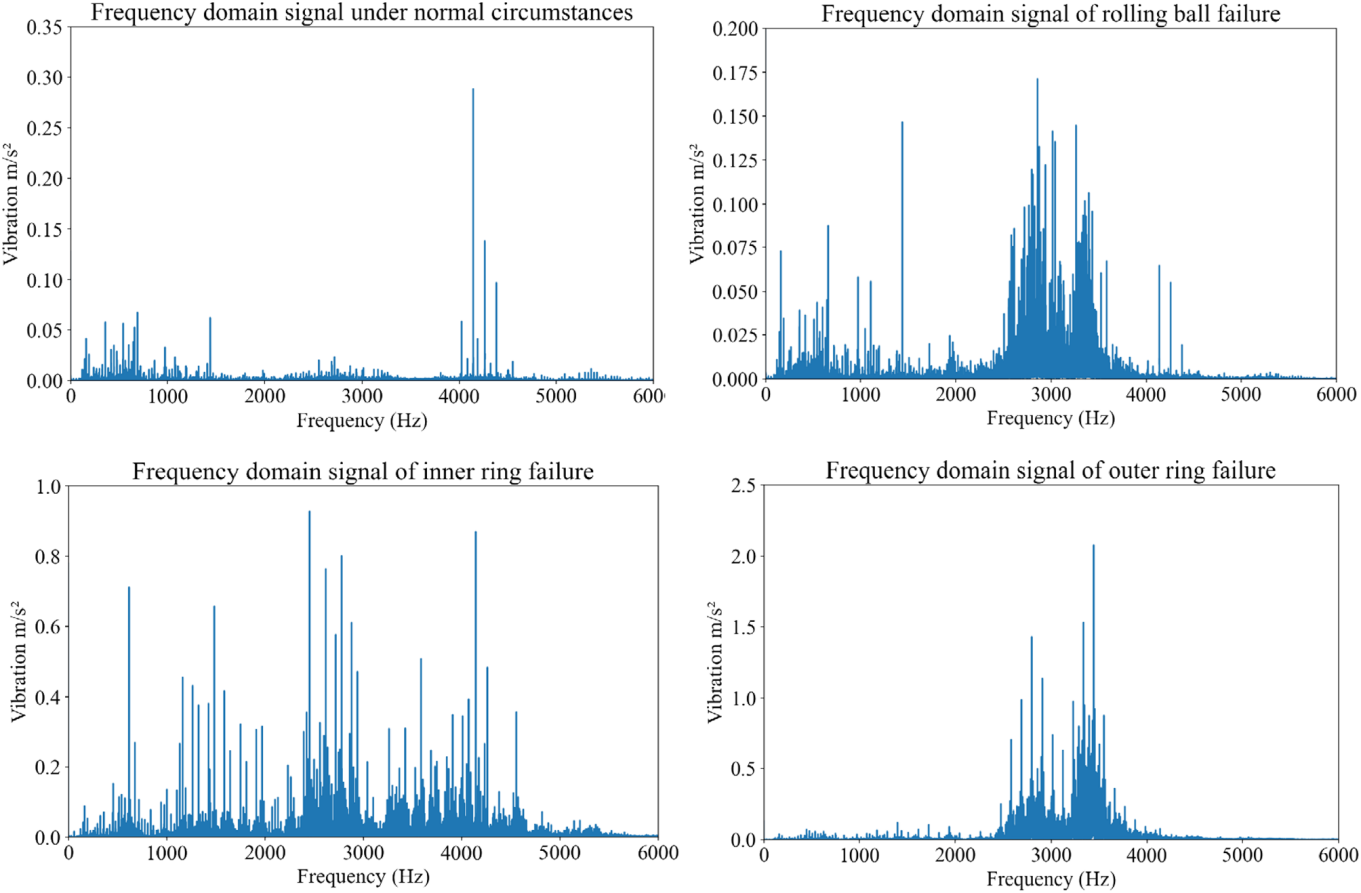
Frequency domain waveform of bearing vibration signal under 0hp working condition.

The fault sample signals were collected by whole cycle sampling of inner ring faults, outer ring faults, and rolling ball faults. The time-domain vibration signal and frequency-domain vibration signal of a certain whole cycle are shown in Figs. 1 and 2, respectively. Usually, diagnostic modes based on vibration signal spectrum analysis are limited by factors such as time–frequency transformation and fault fre-quency dependence on bearing size parameters. Therefore, a more general machine learning method is employed to extract the mean, absolute mean value, waveform factor, peak to peak value, root mean square, standard deviation, skewness, kurtosis, pulse factor, peak factor, autocorrelation coefficient, and variance of bearing fault vi-bration signals. This article extracts 23 feature indicators from the CWRU bearing fault dataset, as shown in Table 1. These feature indicators can be combined with machine learning and fault diagnosis algorithms to classify, predict, and diagnose bearing faults. Generally speaking, researchers can extract suitable features for the analysis and pre-diction of bearing faults based on specific analysis needs and algorithm choices.

**Table 1:** Characteristic indicators for bearing fault diagnosis.

Number	Feature name	Number	Feature name
1	Peak value	13	Wave form
2	Peak-peak value	14	Margin
3	Root-mean-square	15	Center of gravity frequency
4	Average absolute value	16	RMS bandwidth
5	Pulse factor	17	Frequency variance
6	Spectral characteristics	18	Autocorrelation coefficient
7	Energy feature	19	Variance
8	Frequency band energy	20	Standard deviation
9	Frequency peak	21	Wavelet feature mean
10	Skewness	22	Standard deviation of wavelet features
11	Kurtosis	23	Wavelet feature energy value
12	Average value		

## Improved genetic algorithm optimization of BP neural network

### BP neural network

**Figure 3:**
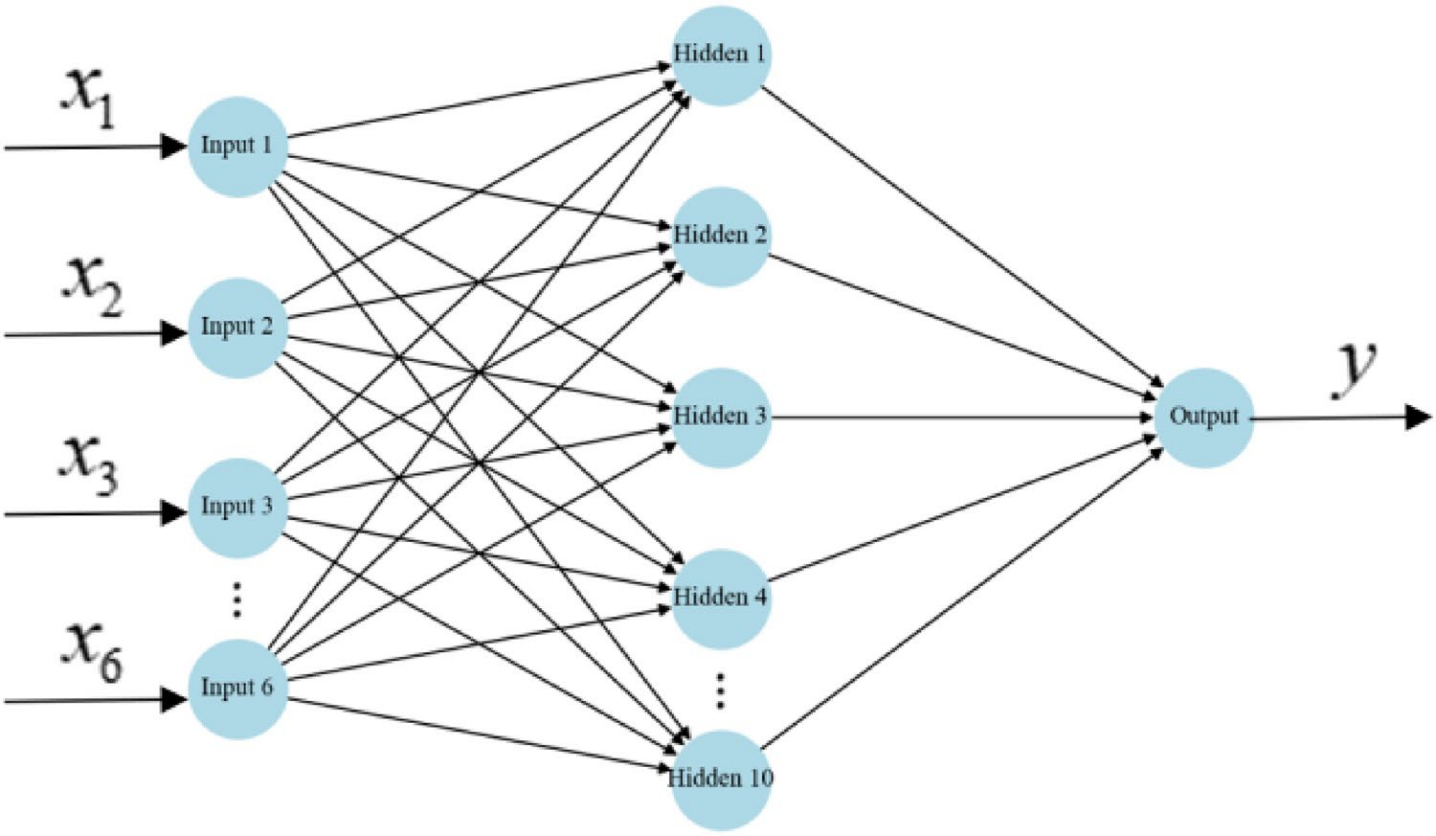
Schematic diagram of neural network topology.

Artificial neural network is a computational model generated by simulating the structure of biological neural networks, which can perform highly nonlinear mapping and has certain stability and fault tolerance. The most widely used among numerous neural network models is the back propagation (BP) neural network model, which is a multi-layer feedforward neural network trained using error backpropagation algorithm. Its model structure consists of an input layer, an output layer, and one or more hidden layers. The topology diagram is shown in Fig. 3, where nodes in the same layer do not interfere with each other and are independent of each other. Nodes in different layers are connected nonlinearly through neural network parameters such as weights and biases. The BP neural network algorithm includes the following two processes:

- Forward propagation of signals.
- Backpropagation of errors.

Assuming the BP network structure with three layers of neurons is $n - q - m$,$x_{1} ,x_{2} , \ldots ,x_{n}$ is the input of the neural network, output as $y_{1} ,y_{2} , \ldots ,y_{m}$.

The commonly used loss functions in BP neural networks are mean squared error (MSE), cross entropy, and so on.

1 $$L_{1} = \frac{1}{m}\sum\limits_{k = 1}^{m} {(y^{\prime}_{k} - y_{k} )^{2} }$$

2 $$L_{2} = - \sum\limits_{i = 1}^{m} {(\widehat{{y_{i1} }}\log y_{i1} + \widehat{{y_{i2} }}\log y_{i2} + \cdots + \widehat{{y_{in} }}\log y_{in} )}$$

Equation (1) represents the mean square error function, and Eq. (2) represents the cross entropy function. In Eq. (1), $y_{k}{\prime}$ represents the predicted value, and $y_{k}$ represents the true value. In Eq. (2), $y_{in}$ represents the predicted sample distribution probability, and $\widehat{y_{in} }$ represents the true sample distribution probability.

If the model output does not meet expectations, the BP neural network will adjust the weights and biases during the error feedback process, achieving adjustments to the computational structure of each neuron and gradually approaching the expected output. In the error correction stage, the weight is often determined using the gradient descent method. Taking the weight correction process of the hidden layer and output layer as an example, the specific mathematical expression is as follows.

3 $$\Delta \omega_{ij} (n + 1) = - \alpha \frac{\partial L}{{\partial \omega_{ij} (n)}}$$

4 $$\omega_{ij} (n + 1) = \omega_{ij} (n) + \Delta \omega_{ij} (n + 1)$$

In formulas (3) and (4), $\alpha$ represents the learning rate, $L$ represents loss.$\Delta \omega_{ij} (n + 1)$ represents the gradient of the loss on the weight $\omega_{ij} (n)$; $\omega_{ij} (n)$ and $\omega_{ij} (n + 1)$ represent the weights before and after the update, respectively.

### Improved genetic algorithm (IGA)

**Figure 4:**
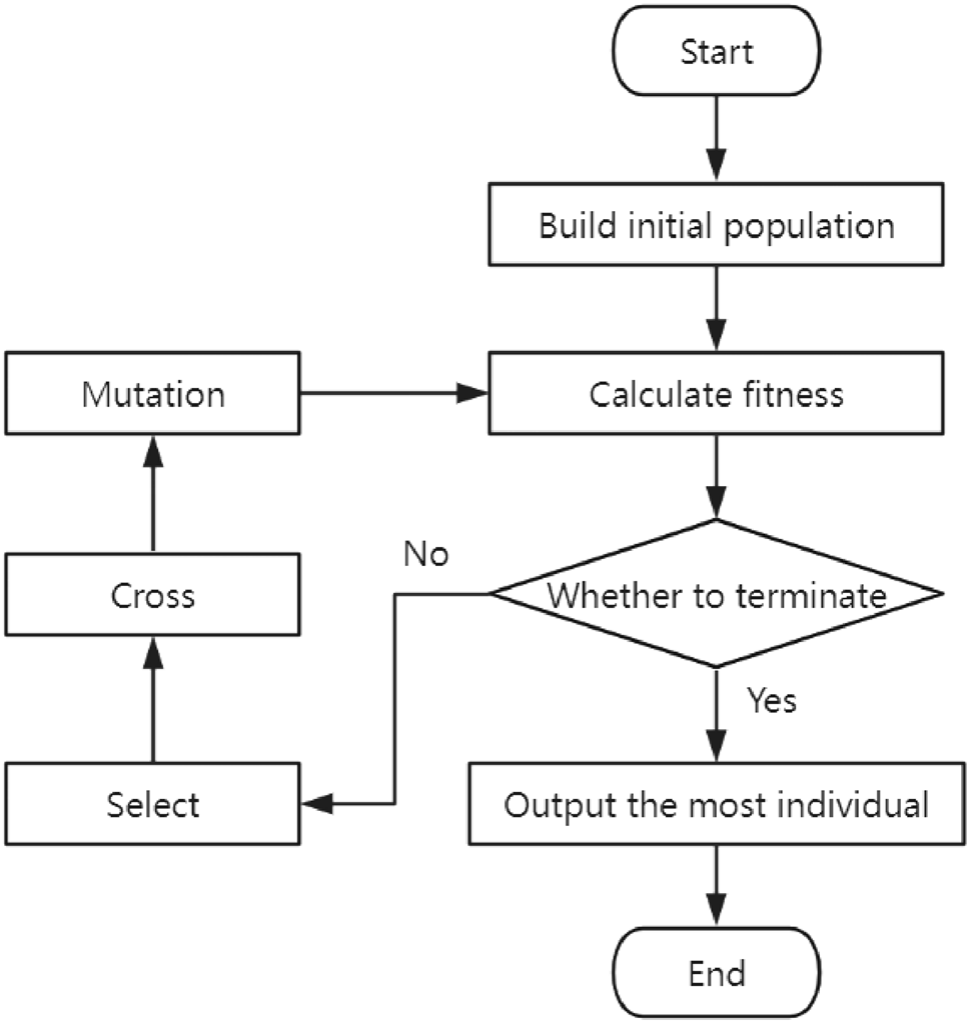
Genetic algorithm operation flowchart.

Genetic algorithm is a stochastic global search optimization method proposed by Professor Holland, which combines Darwin's theory of evolution and Mendel's genetic ideas to solve optimization problems. It has good adaptability and optimization capabilities. The basic concepts of genetic algorithms include chromosome encoding, genes, populations, fitness functions, selection, crossover, and mutation, as well as corresponding operational parameters. Based on the basic idea of survival of the fittest, it simulates the replication, crossover, and mutation phenomena in natural selection and genetics. The genetic operation process is shown in Fig. 4, where the chromosome represents a solution to the problem. Through selection operation, crossover operation, and mutation operation, it evolves to generate individuals that are more suitable for the environment, gradually identifying the optimal solution region. In this way, generation after generation, this group will continue to reproduce and evolve, ultimately converging into a group of individuals that are most suitable for the environment.

The traditional genetic algorithm is a method of finding and solving the optimal solution by simulating the natural process of biological survival of the fittest. However, when dealing with and solving some complex optimization problems, it is easy to fall into some local extreme points. This study proposes an improved Adaptive Genetic Algorithm (IGA) that takes into account the diversity of population fitness and adaptively adjusts the crossover and mutation probabilities of the genetic algorithm in a non-linear manner; In order to improve the convergence efficiency and optimization ability of genetic algorithms, a hierarchical and proportional selection operator is adopted, which not only considers retaining elite individuals but also enhances the diversity of the population.

#### Improved selection operator

The commonly used selection operators include optimal save strategy, random competition selection method, and roulette wheel selection method, all of which are based on the size of individual fitness values for selection. Individuals with higher fitness values are more likely to be selected to enter the next generation, while those with lower fitness values are more likely to be eliminated. However, these traditional selection operators all have some problems to some extent. The roulette wheel selection method will generate random errors, that is, selecting individuals with lower fitness values and eliminating those with higher fitness values. The optimal preservation strategy only focuses on preserving excellent individuals and completely ignores the problem of eliminating individuals with low fitness values, resulting in the problem of local optima. This article improves the selection operator by optimizing its selection mechanism for individuals. While maximizing population diversity, individuals with high fitness values can also be directly inherited to the next generation. The improved selection operator is as follows:

1. Randomly determine the weights (biases) and hyperparameter combination values of a set of neural networks, designate them as the initial population, and calculate the fitness value.
2. Arrange the individuals in the initial population in ascending order based on their fitness values.
3. Select the top 5% and bottom 5% of individuals to skip the crossover stage and directly pass on to offspring, while the remaining population undergoes crossover operations. This can ensure the inheritance of excellent genes from the population to offspring, as well as the diversity of the population.
4. After the crossover is completed, randomly select 15% of individuals to directly enter the offspring.
5. Perform mutation operations on the entire population, randomly selecting 15% of individuals to directly enter offspring.
6. Calculate the fitness value of the entire mutated population and arrange it in ascending order. Divide it into three layers, and randomly select 20% of individuals from each layer to enter the offspring.

#### Improved adaptive crossover and mutation operators

Traditional genetic algorithms use fixed crossover and mutation probabilities, with crossover probabilities typically ranging from 0.3 to 0.7 and mutation probabilities typically ranging from 0.1 to 0.3. But there is a huge problem with this, which is that it is difficult to adjust the probability of crossover operation and mutation operation to the optimal level. If the crossover probability is set too low, the probability of crossover operations occurring decreases, leading to a weakening of the algorithm's exploration ability. Low crossover probability may lead to insufficient communication and combination of genetic information among individuals in the population, making the algorithm trapped in local optima and difficult to discover global optima. If the crossover probability is set too high, the probability of crossover operation occurring may be high, which may lead to the algorithm's excessive dependence on crossover operation, resulting in the generation of new individuals too quickly, and individuals with high fitness values are easily destroyed, which may lead to the algorithm evolving into a random search algorithm. If the mutation probability is set too low, it may lead to the algorithm falling into local optima in the search space and unable to effectively explore the global optimal solution. Low mutation probability may limit the diversity of individual genes, leading to premature convergence or stagnation of the algorithm. If the mutation probability is set too high, it may cause the algorithm to excessively explore the search space and waste computing resources. A high probability of variation may lead to individual genes changing too randomly, making it difficult to maintain high-quality solutions. In addition, a high mutation probability may also cause the algorithm to excessively destroy the already converging high-quality solutions and fall into an unstable state. In response to the various shortcomings of traditional genetic algorithms, many scholars have continuously made improvements and proposed many improved algorithms, such as Adaptive Genetic Algorithm (AGA), which is shown in Eqs. (8) and (9)^26^.

5 $$P_{c} = \left\{ {\begin{array}{*{20}c} {\frac{{k_{1} (f_{\max } - f^{\prime})}}{{f_{\max } - f_{avg} }},\;\;\;\;\;\;\;\;\;\;\;\;\;\;f^{\prime} \ge f_{avg} } \\ {k_{2} \;\;\;\;\;\;\;\;\;\;\;\;\;\;\;\;\;\;\;\;\;\;\;\;\;\;\;\;\;\;f^{\prime} < f_{avg} } \\ \end{array} } \right.$$

6 $$P_{m} = \left\{ {\begin{array}{*{20}c} {\frac{{k_{3} (f_{\max } - f^{\prime})}}{{f_{\max } - f_{avg} }},\;\;\;\;\;\;\;\;\;\;\;\;\;\;f \ge f_{avg} } \\ {k_{4} \;\;\;\;\;\;\;\;\;\;\;\;\;\;\;\;\;\;\;\;\;\;\;\;\;\;\;\;\;\;f < f_{avg} } \\ \end{array} } \right.$$

In formulas (5) and (6), $f^{\prime}$ represents the larger fitness value among the two individuals to be crossed; $f_{\max }$ is the maximum fitness in the population; $f_{avg}$ is the average fitness of the population; $f$ represents the fitness of the current individual; $k_{1}$-$k_{4}$ are adaptive control parameters. However, the AGA algorithm evolves slowly in the early stages and is prone to stagnation. Excellent individuals are basically in a stationary state, that is, there is no crossover or mutation operation. In the later stage, the probability of crossover and mutation tends to stabilize. Ultimately, individuals with high fitness values in the population are likely to converge locally, thus failing to achieve global optimization, which can easily lead genetic algorithms to evolve towards local convergence.

To retain elite individuals and avoid the population falling into local optima, this paper introduces the Tanh activation function to improve the adaptive crossover probability and mutation probability, using adjusted crossover probability $P_{c}$ and mutation probability $P_{m}$, as shown in formulas (7) to (8).

7 $$P_{c} = \left\{ {\begin{array}{*{20}c} {P_{c\_\min } + (P_{c\_\max } - P_{c\_\min } )\frac{{e^{{f_{i} - f_{iavg} }} - e^{{ - (f_{i} - f_{iavg} )}} }}{{e^{{f_{i} - f_{iavg} }} + e^{{ - (f_{i} - f_{iavg} )}} }},\;\;\;\;f_{i} \ge f_{avg} } \\ {p_{c\_\max \;\;\;\;\;\;\;\;\;\;\;\;\;\;\;\;\;\;\;\;\;\;\;\;\;\;\;\;\;\;\;\;\;\;\;\;\;\;\;\;\;\;\;\;\;\;\;\;\;\;\;\;\;\;\;\;\;\;\;\;\;\;\;\;\;\;\;\;} f_{i} < f_{avg} } \\ \end{array} } \right.$$

8 $$P_{m} = \left\{ {\begin{array}{*{20}c} {P_{m\_\min } + (P_{m\_\max } - P_{m\_\min } )\frac{{e^{{f_{i} - f_{iavg} }} - e^{{ - (f_{i} - f_{iavg} )}} }}{{e^{{f_{i} - f_{iavg} }} + e^{{ - (f_{i} - f_{iavg} )}} }},\;\;\;\;f_{i} \ge f_{avg} } \\ {p_{m\_\max \;\;\;\;\;\;\;\;\;\;\;\;\;\;\;\;\;\;\;\;\;\;\;\;\;\;\;\;\;\;\;\;\;\;\;\;\;\;\;\;\;\;\;\;\;\;\;\;\;\;\;\;\;\;\;\;\;\;\;\;\;\;\;\;\;\;\;\;} f_{i} < f_{avg} } \\ \end{array} } \right.$$

In the above equation, $P_{s\_\max }$, $P_{s\_\min }$, $P_{c\_\max }$, $P_{c\_\min }$, $P_{m\_\max }$, and $P_{m\_\min }$ represent the maximum and minimum values of selection probability, cross probability, and mutation probability, respectively. $f_{i}$ represents the fitness of individuals in the $i$-th generation population, and $f_{iavg}$ represents the average fitness of the $i$-th generation population.

### IGA-BPNN algorithm based on IGA optimization for BP neural network

This paper refers to the connection weights and biases of BP neural networks as weight parameters, referred to as neural network parameter one, and the training hyperparameters of BP neural networks as neural network parameter two, collectively referred to as dual parameter combinations. The method proposed in this paper is to use an improved genetic algorithm to optimize the weight parameters of the BP neural network, in order to solve the problems of slow convergence speed and low accuracy caused by the random initial weights of the BP neural network. In addition, due to the poor optimization performance of existing neural network hyperparameter optimization algorithms, they are prone to getting stuck in local optima, resulting in large network model errors that are difficult to meet practical application needs; Moreover existing neural network hyperparameter optimization algorithms have slow convergence speed and high time cost. This paper combines an improved genetic algorithm with a BP neural network for training and solving, in order to efficiently find the optimal BP neural network weight and hyperparameter—dual parameter combination, in order to solve the problems of slow convergence speed and easy falling into local optimal solutions.

#### Chromosome coding and parameter initialization

Due to the fact that the dataset used in this article is all floating-point numbers, the accuracy of model prediction is required to be high, and the algorithm is also expected to have good stability. Therefore, real number encoding is used to encode chromosomes, with the length of chromosome encoding being the sum of the number of weights and hyperparameters, and the initial value of chromosomes is set to be equal to the random points in the search space. Assuming that the BP neural network model adopts a unified network topology structure, the model consists of one input layer, three hidden layers, and one output layer. The number of neuron nodes in the input layer of the network is equal to the dimension of the input data sample, the hidden layer contains three fully connected layers, and the output layer contains one node, representing the network output.

Choosing an appropriate range of hyperparameters is a crucial step in ensuring that the network model can fully learn and provide optimal performance. This article combines relevant literature research and work experience, as well as conducts preliminary tests on different combinations of hyperparameters. Considering the constraints of computing resources, the following range of hyperparameters is set.Let the number of neurons in the 1–3 hidden layers be $n_{1}$, $n_{2}$, and $n_{3}$. The activation functions used in the three hidden layers are represented by symbols $a_{1}$, $a_{2}$, and $a_{3}$, with values taken as integers within the interval [0,5], corresponding to six types of activation functions: ReLU, Sigmoid, Softmax, tanh, Softplus, and Softsign. During training, the number of samples selected for a single training batch is usually set to 32, 64, 128, 256, represented by integers within the interval [0,3]. The optimizer uses [0,4] to represent SGD, Adam, RMSpro, Adagrad, and Adadelta, respectively. Training generations use [0,5] to represent 100, 200, 300, 400, 500, and 600, respectively. The loss function uses [0,3] to represent mean square error(MSE), mean absolute error(MAE), classification cross entropy(CCE), and logarithmic variance(LV), respectively. The learning rate lr usually takes values of 0.01, 0.001, 0.0001, and is represented by integers within the interval of [0, 2]. The range of hyperparameter values is shown in Table 2.

**Table 2:** Range of hyperparameter values for neural networks.

Hyperparameter name	Value range
Activation function $a_{1}$	[0,5]
Activation function $a_{2}$	[0,5]
Activation function $a_{3}$	[0,5]
Batch	[0,3]
Optimizer	[0,4]
Training generation	[0,5]
Loss function	[0,3]
Learning rate(lr)	[0,2]

The length of the chromosome is $l = (n + 1) * n_{1} + (n_{1} + 1) * n_{2} + (n_{2} + 1) * n_{3} + (n_{3} + 1) * m + 8$, where $n$ represents the number of neurons in the input layer and $m$ represents the number of neurons in the output layer.

#### Configuration of fitness function for IGA

The fitness function of genetic algorithms is a criterion used to evaluate the quality of individuals. Due to the fact that genetic algorithms are essentially independent of external information in evolutionary search, a reasonable fitness function $f$ should be selected as the sole criterion for evaluation. Here, the fitness function uses the mean absolute error (MAE) of the network model on the dataset, represented as:

9 $$f = MAE = \frac{{\sum\limits_{i = 1}^{n} {\left| {y^{\prime}_{i} - y_{i} } \right|} }}{n}$$

In formula (9), $y^{\prime}_{i}$ and $y_{i}$ respectively represent the predicted and true values of the sample data, where $n$ represents the number of samples.

#### Implementation process of IGA-BPNN algorithm

**Figure 5:**
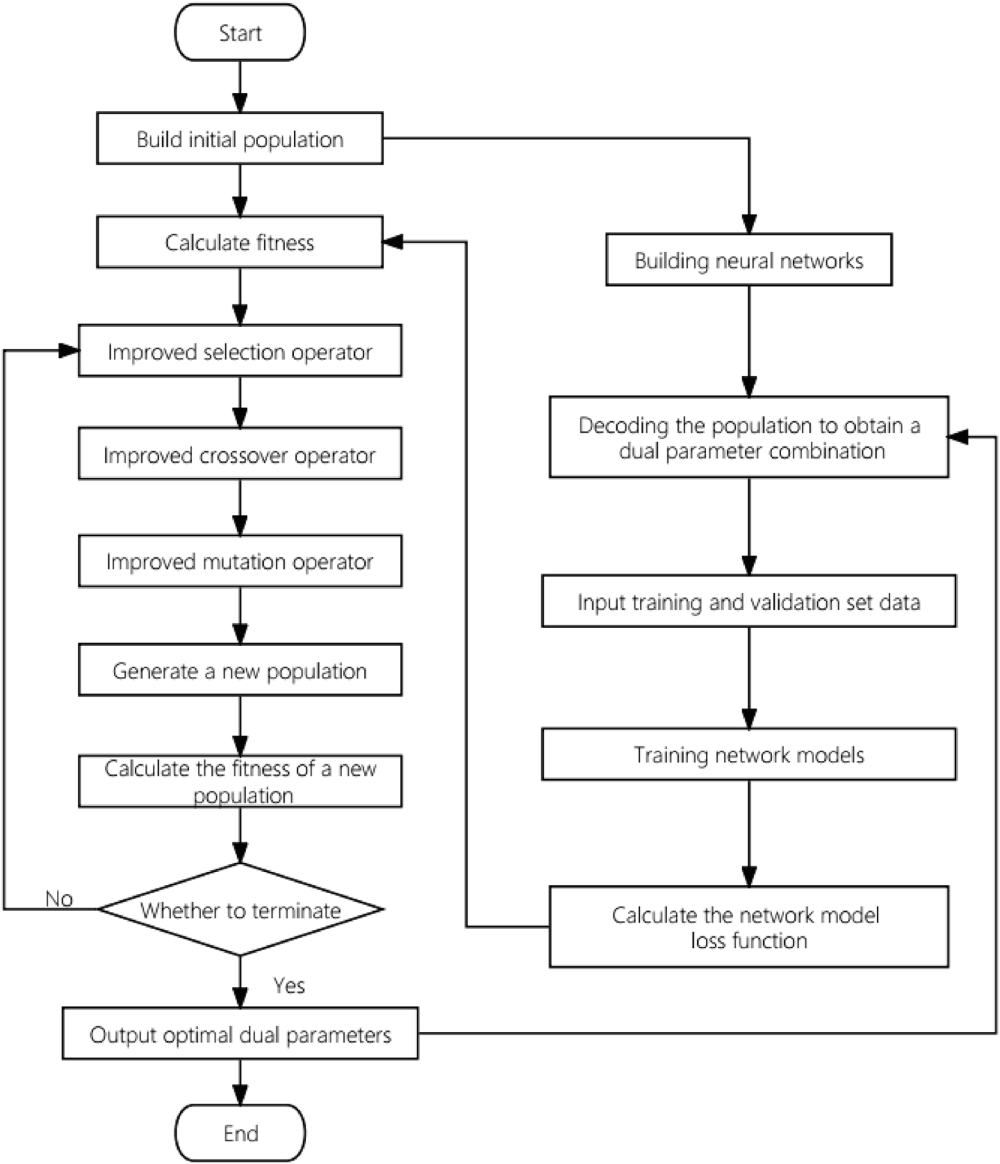
IGA-BPNN algorithm flowchart.

This article proposes an algorithm IGA-BPNN that utilizes an improved genetic algorithm to automatically optimize the weights and hyperparameters of BP neural networks. This method is based on neural networks. Firstly, the topology structure of the neural network and the hyperparameters that need to be optimized are determined, and at the same time, the upper and lower bounds of the parameter space of the hyperparameters are determined; Then, a combination of NP group weights and hyperparameters is randomly generated (NP is the population size), and each combination is encoded as a chromosome as input to the IAG-BPNN algorithm. The IGA-BPNN algorithm uses the fitness function shown in Eq. (11) to continuously select, cross, and vary, in order to obtain the individual which has the best fitness; Finally, the chromosome is decoded to obtain a set of weights and hyperparameters that optimize the fitness. Finally, the neural network is trained using the combination of these weights and hyperparameters. The entire implementation process of the IGA-BPNN algorithm is shown in Fig. 5.

The IGA-BPNN for weight and hyperparameter optimization algorithm proposed in this article is described in Algorithm 1.

Algorithm 1: IGA-BPNN for weight and hyperparameter optimization algorithm.

Input: Neural network topology, range of hyperparameter values for neural networks, population size NP for IGA-BPNN algorithm, maximum number of iterations N.

Output: The optimal set of weights and neural network hyperparameters.

- Initialize the neural network topology and determine the parameter space of the neural network hyperparameters;
- Initialize the genetic algorithm population P(0), population size NP, maximum number of iterations N, crossover probability $P_{c}$, mutation probability $P_{m}$, etc.;
- While (maximum number of iterations not reached):
- Calculate the fitness $f_{i}$ of each individual, decode a set of weights and hyperparameters corresponding to the BP neural network model for each individual, and use the average absolute error of the model on the training set as the fitness $f_{i}$;
- Using an improved hierarchical proportional selection operator to select individuals as parents from the population;
- Using a two-point crossover operator with improved crossover probability to perform crossover operations on the parent generation, generating new individuals;
- Using a single point mutation operator with improved mutation probability to perform mutation operations on the crossed offspring, selecting offspring to form a new population according to the improved selection operator;
- End while;
- Decoding the globally optimal individual to obtain the optimal set of weights and hyperparameter combinations;
- Train the BP neural network using the optimal combination of weights and hyperparameters.

## Experimental results

### Evaluating indicator

In order to verify the effectiveness of the IGA-BPNN algorithm proposed in this paper, the algorithm that only optimizes neural network weights proposed in this paper is defined as the IGA-BPNN-1 algorithm, and the algorithm that only optimizes neural network hyperparameters proposed in this paper is defined as the IGA-BPNN-2 algorithm. The algorithm that optimizes neural network weights and hyperparameters using traditional genetic algorithms is defined as GA-BPNN, the algorithm that only optimizes neural network weights using traditional genetic algorithms is defined as GA-BPNN-1 algorithm, and the algorithm that only optimizes neural network hyperparameters using traditional genetic algorithms is defined as GA-BPNN-2 algorithm. Simultaneously, an unoptimized BPNN algorithm is introduced as the baseline to compare the performance and efficiency of these seven algorithms. The above 7 methods all use the features extracted in this article for training and prediction. Then, the method proposed in paper^27^ is used to extract features, and the improved algorithm IGA-BPNN proposed in this paper is used for training and prediction, which is defined as IGA-BPNN-27. Finally, the method proposed in paper^27^ was used to extract features through experiments using CNN and LSTM models. The experimental results showed that the IGA-BPNN method proposed in this paper still has significant advantages in computational efficiency and fault diagnosis performance.

In order to comprehensively evaluate the accuracy of the prediction results of each model, mean square error (RMSE), mean absolute error (MAE), and cross entropy loss were used to compare and analyze the performance ability of each model. The specific calculation formula for each indicator is as follows:

10 $$MSE = \frac{{\sum\limits_{i = 1}^{n} {(y^{\prime}_{i} - y_{i} )^{2} } }}{n}$$

11 $$MAE = \frac{{\sum\limits_{i = 1}^{n} {\left| {y^{\prime}_{i} - y_{i} } \right|} }}{n}$$

12 $$loss = \sum\limits_{i = 1}^{n} y_{i} \log (y_{i}^{\prime} )$$

In Eqs. (10), (11), and (12), $y^{\prime}_{i}$ and $y_{i}$ represent the predicted and true values of the sample data, where $n$ represents the number of samples.

Due to the different weighting, threshold, hyperparameter assignment methods, and training functions of neural network models, their predictive performance also varies. In order to more accurately reflect the differences in prediction performance of different models, this article trains each model 20 times and conducts statistical analysis on four evaluation indicators: MSE, MAE, cross entropy loss, and total number of iterations calculated for each prediction. The prediction performance of the models is compared.

### Dataset of bearing fault

The following experimental comparative analysis will be conducted based on the feature indicators extracted in this article and the feature indicators extracted in the method described in paper^27^, as shown in Tables 3 and 4, respectively.

**Table 3:** The sample dataset of this article.

Sample type	Characteristic length	Number of samples			Sample labels
		Training set	Validation set	Test set	
NORMAL	38	6000	2000	2000	0
OR021	38	6000	2000	2000	1
OR014	38	6000	2000	2000	2
OR007	38	6000	2000	2000	3
IR021	38	6000	2000	2000	4
IR014	38	6000	2000	2000	5
IR007	38	6000	2000	2000	6
B021	38	6000	2000	2000	7
B014	38	6000	2000	2000	8
B007	38	6000	2000	2000	9

**Table 4:** Sample dataset for paper^27^.

Sample type	Characteristic length	Number of samples			Sample labels
		Training set	Validation set	Test set	
NORMAL	864	6000	2000	2000	0
OR021	864	6000	2000	2000	1
OR014	864	6000	2000	2000	2
OR007	864	6000	2000	2000	3
IR021	864	6000	2000	2000	4
IR014	864	6000	2000	2000	5
IR007	864	6000	2000	2000	6
B021	864	6000	2000	2000	7
B014	864	6000	2000	2000	8
B007	864	6000	2000	2000	9

Based on the previous discussion, this study set 23 factors that affect the detection results of bearing faults as feature indicators for the model, and used a BP neural network to determine the rationality of their settings. The 23 input factors of the neural network are peak value, peak to peak value, root mean square value, average absolute value, pulse factor, spectral feature, energy feature, frequency band energy, frequency peak value, skewness, kurtosis, waveform, margin, center of gravity frequency, etc. Root mean square bandwidth, frequency variance, autocorrelation coefficient, variance, and wavelet features. By defining the number of wavelet feature decomposition levels in Table 1 as 5, we can ultimately obtain 18 wavelet features, and with the addition of an additional 20 features, we can ultimately obtain 38 features in this paper. In the above dataset, NORMAL represents the normal condition of the bearing during operation, represented by label 0. OR021 represents an outer ring fault with a diameter of 21 mils, represented by label 1. OR014 represents a fault in the outer ring with a diameter of 14 mils, indicated by label 2. OR007 represents an outer ring fault with a diameter of 7 mils, indicated by label 3. IR021 represents an inner ring fault with a diameter of 21 mils, indicated by label 4. IR014 represents an inner ring fault with a diameter of 14 mils, indicated by label 5. IR007 represents an inner ring fault with a diameter of 7 mils, indicated by label 6. B021 represents a rolling element fault with a diameter of 21 mils, indicated by label 7. B014 represents a rolling element fault with a diameter of 14 mils, indicated by label 8. B007 represents a rolling element fault with a diameter of 7 mils, indicated by label 9. At the same time, the above dataset is preprocessed to extract features, and fault diagnosis models are established using the aforementioned algorithms for training and prediction.

**Figure 6:**
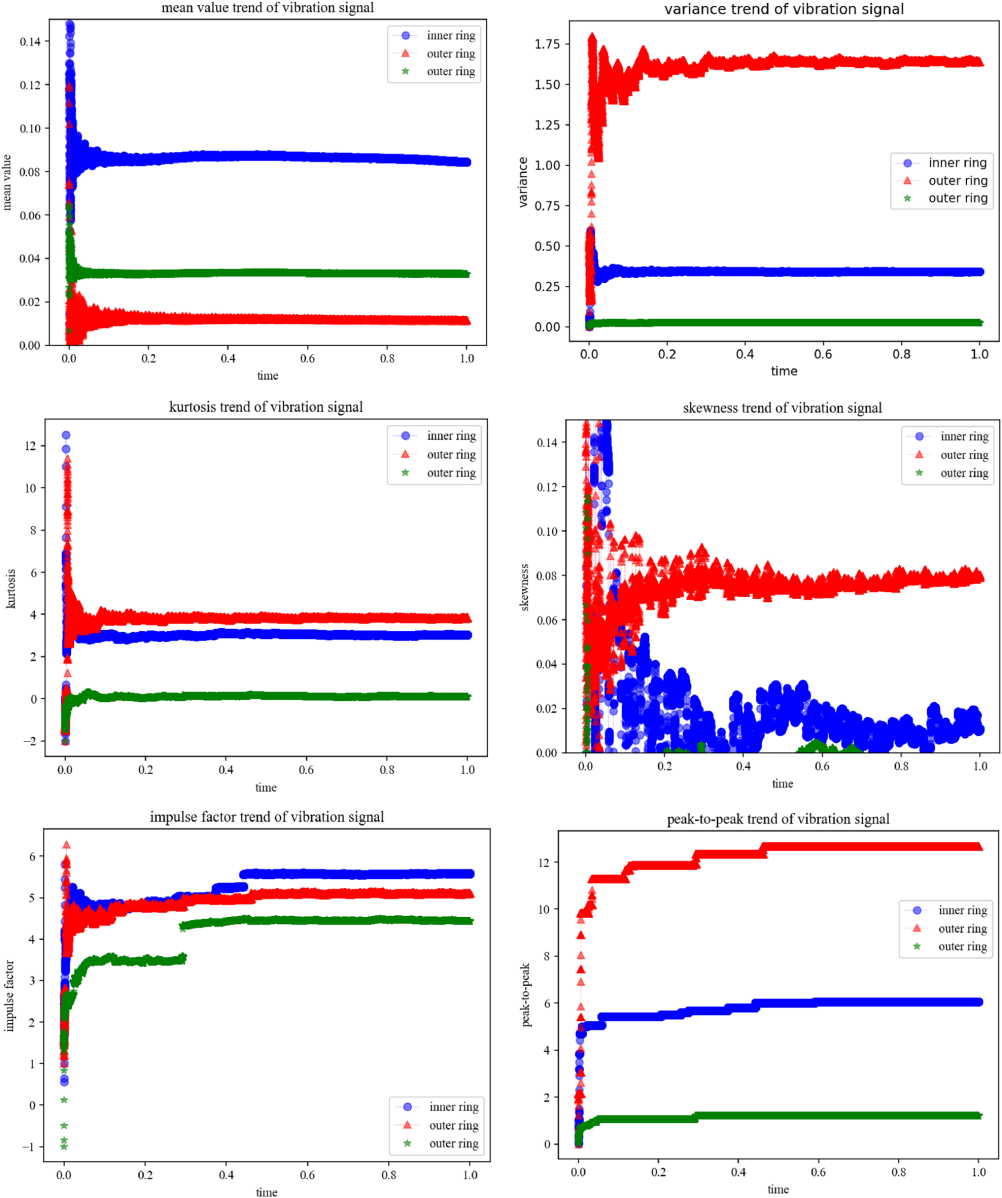
Trend of variation of different characteristic values under different faults.

Due to space limitations in the article, as shown in Fig. 6, the waveform diagram below shows features such as mean, variance, kurtosis, skewness, pulse factor, and peak to peak value. From Fig. 6, it can be seen that the different signal characteristic parameters extracted after analysis and processing can clearly distinguish different types of faults from the original vibration signal shown in Figs. 1 and 2.

### Analysis of error

In order to quantitatively analyze the advantages and disadvantages of the 8 methods mentioned above, 20 independent repeated experiments were conducted on the above dataset. The IGA-BPNN algorithm proposed in this paper was compared with the bearing fault diagnosis models established by IGA-BPNN-1, IGA-BPNN-2, GA-BPNN-1, GA-BPNN-2, BPNN, and IGA-BPNN-27, respectively. The average absolute error (MAE) and mean square error (MSE) were used as evaluation indicators for the model. The MAE comparison of the fault diagnosis models established by these eight algorithms is shown in Table 5, and the MSE comparison is shown in Table 6. From Tables 5 and 6, it can be seen that the MAE and MSE of the IGA-BPNN algorithm are superior to other algorithm models.

**Table 5:** MAE comparison of eight algorithms.

Algorithm	Optimal value /10–2	Worst value /10–2	Average value/10^–2^
IGA-BPNN	0.2397	2.6862	1.0903
IGA-BPNN-1	0.2668	2.8713	1.1159
IGA-BPNN-2	0.2684	2.8773	1.1291
GA-BPNN	0.2671	3.6727	1.1650
GA-BPNN-1	0.2612	3.7674	1.1649
GA-BPNN-2	0.2679	3.7698	1.1668
BPNN	0. 2792	4.1352	1.3162
IGA-BPNN-27	0. 2702	4.2117	1.2344

**Table 6:** MSE comparison of eight algorithms.

Algorithm	Optimal value /10–2	Worst value /10–2	Average value /10^–2^
IGA-BPNN	0.0832	0.2415	0.1381
IGA-BPNN-1	0.0974	0.2786	0.1754
IGA-BPNN-2	0.1001	0.2843	0.1812
GA-BPNN	0.1130	0.2776	0.1751
GA-BPNN-1	0.1232	0.2749	0.1741
GA-BPNN-2	0.1291	0.2782	0.1759
BPNN	0.1326	0.2706	0.1877
IGA-BPNN-27	0.1016	0.2320	0.1526

### Analysis of algorithm convergence speed

**Figure 7:**
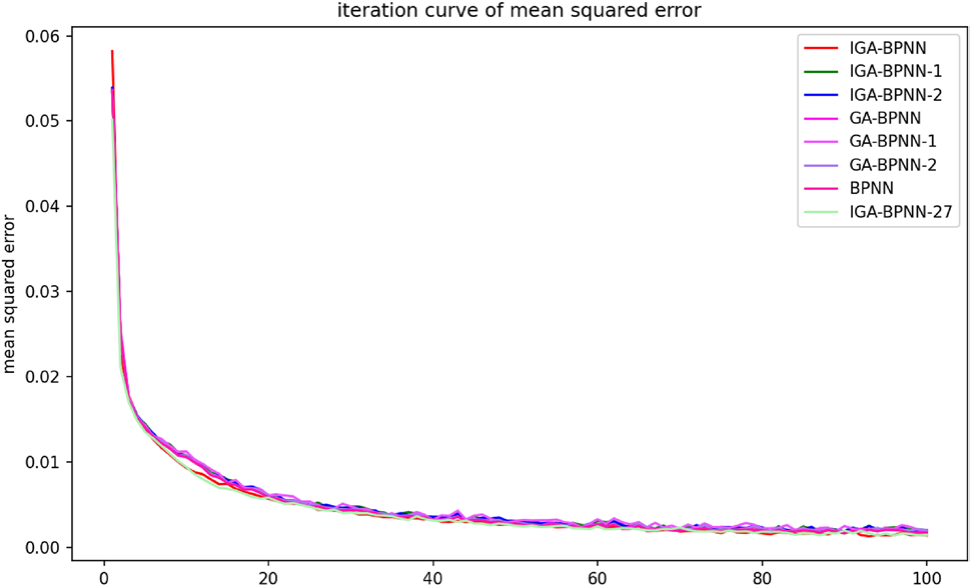
Iterative curves of mean square error for eight algorithms.

This article uses IGA-BPNN to train and predict bearing fault diagnosis models with eight algorithms: IGA-BPNN-1, IGA-BPNN-2, GA-BPNN-1, GA-BPNN-2, BPNN, and IGA-BPNN-27. In order to compare their convergence rates, the most representative experiment is selected from them, and its convergence algebra is recorded. At the same time, its convergence curve is plotted. Among them, the MSE convergence curves of the network model for eight algorithms during the optimization process are shown in Fig. 7, and the corresponding convergence algebraic analysis results are listed in Table 7.

**Table 7:** Convergence algebra of eight algorithms.

Algorithm	Convergent algebra	MSE/10–2
IGA-BPNN	65	0. 1352
IGA-BPNN-1	68	0.1615
IGA-BPNN-2	69	0.1622
GA-BPNN	67	0.1679
GA-BPNN-1	68	0.1642
GA-BPNN-2	69	0.1691
BPNN	70	0.1706
IGA-BPNN-27	69	0.1586

**Figure 8:**
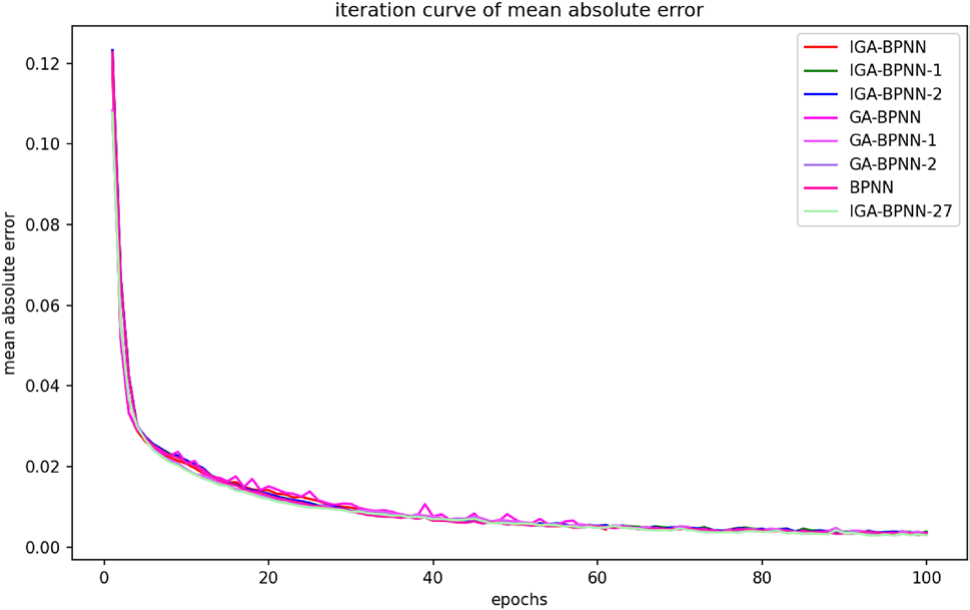
Iterative curves of the mean absolute error of eight algorithms.

**Figure 9:**
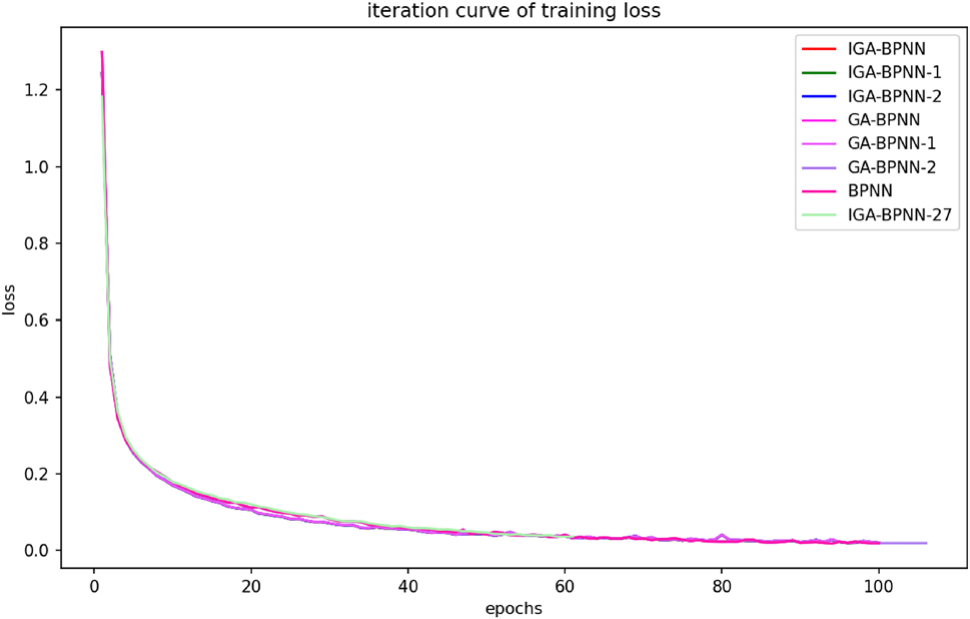
Iterative curves of training loss for eight algorithms.

**Figure 10:**
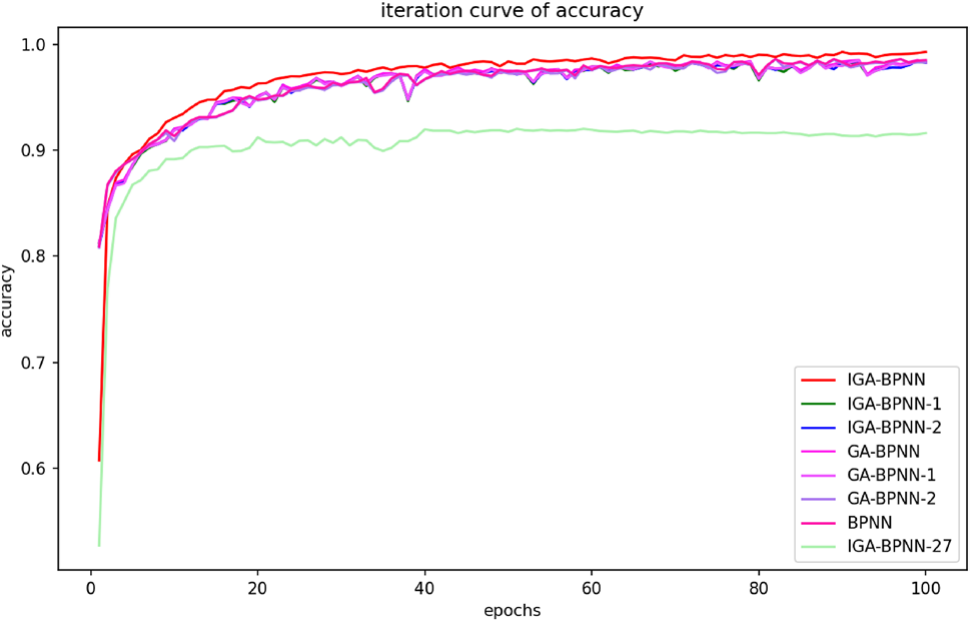
Iterative curves of accuracy for eight algorithms.

According to the results in Figs. 7, 8, 9 and 10 and Table 7, it can be clearly observed that the IGA-BPNN algorithm proposed in this paper has the lowest convergence algebra, followed by the GA-BPNN algorithm, and the IGA-BPNN algorithm also achieved the minimum mean square error. This indicates that the IGA-BPNN algorithm performs well in optimization and has a good convergence speed.

Through quantitative analysis, it can be seen that the proposed algorithm IGA-BPNN reduces the number of convergence iterations by 4.62% compared to IGA-BPNN-1; Compared with IGA-BPNN-2, the number of convergence iterations has been reduced by 6.15%; Compared with GA-BPNN, the number of convergence iterations has decreased by 3.08%; Compared with GA-BPNN-1, the number of convergence iterations has decreased by 4.62%; Compared with GA-BPNN-2, the number of convergence iterations has been reduced by 6.15%; Compared with BPNN, the number of convergence iterations has decreased by 7.69%; Compared with IGA-BPNN-27, the number of convergence iterations has decreased by 6.15%.

By observing Figs. 7, 8, 9 and 10 and Table 7, it can be intuitively observed that IGA-BPNN has the fastest convergence speed and the obtained mean square error(MSE) is also close to optimal. In summary, under limited time and iteration times, the algorithm proposed in this paper can maintain a good level of convergence speed and error loss compared to the other seven BP neural network algorithms for the bearing fault diagnosis model.

### Comprehensive diagnostic analysis

**Figure 11:**
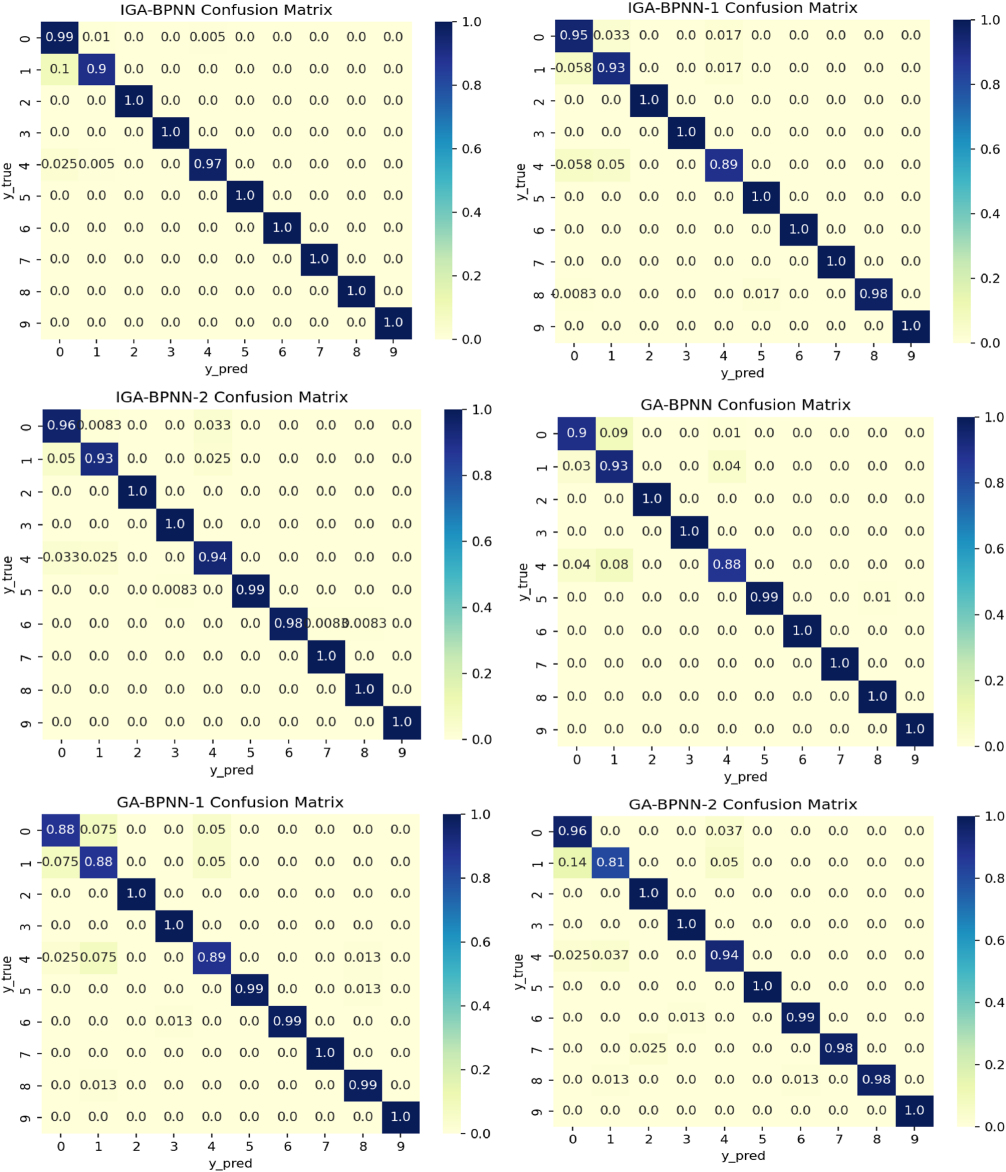

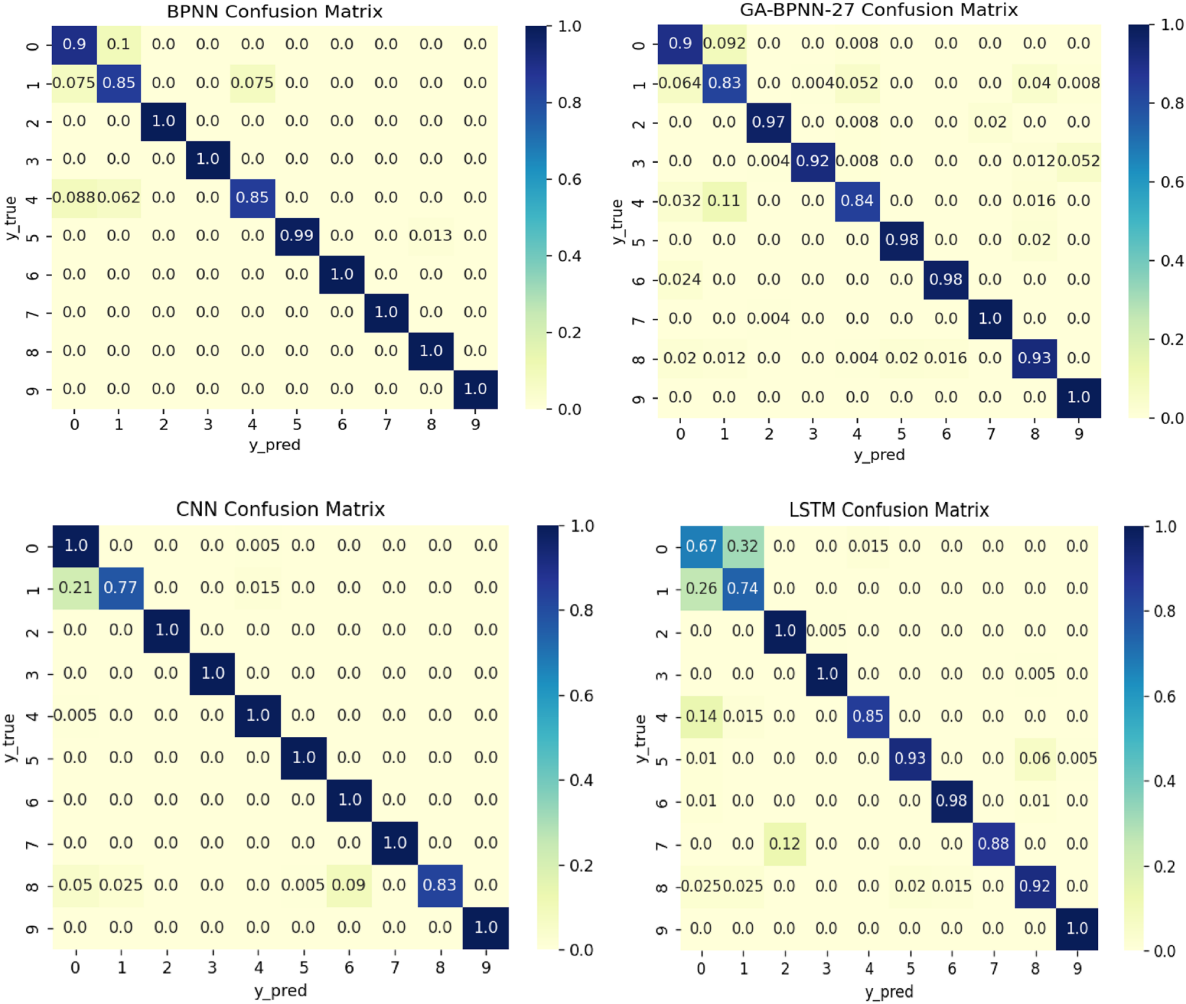
Confusion matrix of eight bearing fault diagnosis models.

As mentioned above, the population size of the genetic algorithm in the seven fault diagnosis models used in this article, IGA-BPNN, IGA-BPNN-1, IGA-BPNN-2, GA-BPNN-27, etc., is set to 200, chromosome length is set to 52, population iteration number is set to 100, $P_{c\_\min }$ is set to 0.2, $P_{c\_\max }$ is set to 0.8, $P_{m\_\min }$ is set to 0.2, and $P_{m\_\max }$ is set to 0.8. In addition, since the BPNN algorithm does not use genetic algorithms, the loss function of the BPNN algorithm is set to the cross entropy function categorical, the optimizer is set to Adam, and the learning rate is set to 0.01. Obtain the confusion matrix of eight algorithm experimental results, as shown in Fig. 11. In the confusion matrix, row labels are predicted labels and column labels are true labels. The darker the color of the diagonal blocks, the higher the corresponding classification accuracy, while the lighter the color of the remaining blocks, the better. From Fig. 11, it can be seen that the IGA-BPNN model proposed in this article not only achieves high diagnostic accuracy in fault diagnosis, but also performs excellently in diagnostic accuracy. This shows the superiority of the IGA-BPNN model in bearing fault diagnosis.

According to the IGA-BPNN fault diagnosis model, a confusion matrix of classification results was extracted from the dataset in this article. Table 5 lists the classification accuracy, precision, recall, and comprehensive evaluation index (F1) for each type of diagnostic model to evaluate the bearing fault diagnosis effectiveness of the eight models. The fault diagnosis results of each neural network model are shown in Table 8.

**Table 8:** Classification index data of experimental sample.

Algorithm	Accuracy	Precision	Recall	F-score
IGA-BPNN	0.9855	0.9879	0.9855	0.9867
IGA-BPNN-1	0.9733	0.9779	0.9733	0.9751
IGA-BPNN-2	0.9683	0.9778	0.9683	0.9729
GA-BPNN	0.9717	0.9778	0.9717	0.9743
GA-BPNN-1	0.9650	0.9709	0.9650	0.9678
GA-BPNN-2	0.9610	0.9676	0.9610	0.9632
BPNN	0.9587	0.9679	0.9588	0.9628
IGA-BPNN-27	0.9128	0.9233	0.9128	0.9157
CNN	0.9590	0.9670	0.9590	0.9599
LSTM	0.8805	0.9109	0.8805	0.8933

From the data in the table, it can be seen that the accuracy, recall, and comprehensive evaluation index F1 score of the IGA-BPNN model, IGA-BPN-1 model, and IGA-BPN-2 model used for bearing fault diagnosis are significantly higher than those of other algorithm models except GA-BPNN algorithm. The IGA-BPNN model has the best fault diagnosis performance, with all indicators higher than other models, reaching 98.55%, 98.79%, 98.55%, and 98.67%, respectively.

From the above classification index data, it can be seen that all BPNN diagnostic models optimized by genetic algorithm have better prediction results than traditional unoptimized BPNN models. The GA-BPNN algorithm has better diagnostic performance than GA-BPNN-1 and GA-BPNN-2 models that only optimized some parameters due to the use of genetic algorithms to optimize the combination of neural network weights and hyperparameters. The IGA-BPNN algorithm has improved the selection, crossover, and mutation operators of genetic algorithms, and its predictive performance is also better than the unimproved GA-BPNN model. Although the GA-BPNN model did not improve the genetic algorithm, the use of genetic algorithm to optimize the dual parameter combination of BPNN neural network weights and hyperparameters resulted in better predictive performance than the diagnostic models GA-BPNN-1 and GA-BPNN-2, which only used a single parameter combination. Although the IGA-BPNN-27 model used an improved genetic algorithm to optimize the combination of neural network weights and hyperparameters, it did not adopt the feature extraction rules proposed in this paper, resulting in lower diagnostic accuracy for bearing faults compared to other fault diagnosis models. Similarly, due to the absence of the feature extraction rules proposed in this article and the lack of improvements to traditional CNN and LSTM algorithms, only the vibration signals extracted by the method proposed in paper^27^ were input into the traditional CNN and LSTM models for fault diagnosis, resulting in lower accuracy in bearing fault diagnosis compared to the BP neural network model optimized by genetic algorithm.This indicates that the feature extraction method proposed in this paper has a significant effect on improving diagnostic accuracy.

In summary, the IGA-BPNN model used in this paper has superior performance in bearing fault diagnosis by preprocessing the vibration signals of the Case Western Reserve University bearing fault dataset and extracting features from the dataset through wavelet packet transform and other methods. Using the IGA algorithm to optimize the weights and hyperparameter combinations of BPNN networks not only eliminates the extremely time-consuming task of manual tuning, but also enables faster selection of the most suitable initial weights and hyperparameter combinations for the network, thereby obtaining more accurate and precise results of fault diagnosis. In summary, the IGA-BPNN model proposed in this article has stable classification performance when facing complex data, which can ensure good fault diagnosis performance.

### Volatility analysis

**Figure 12:**
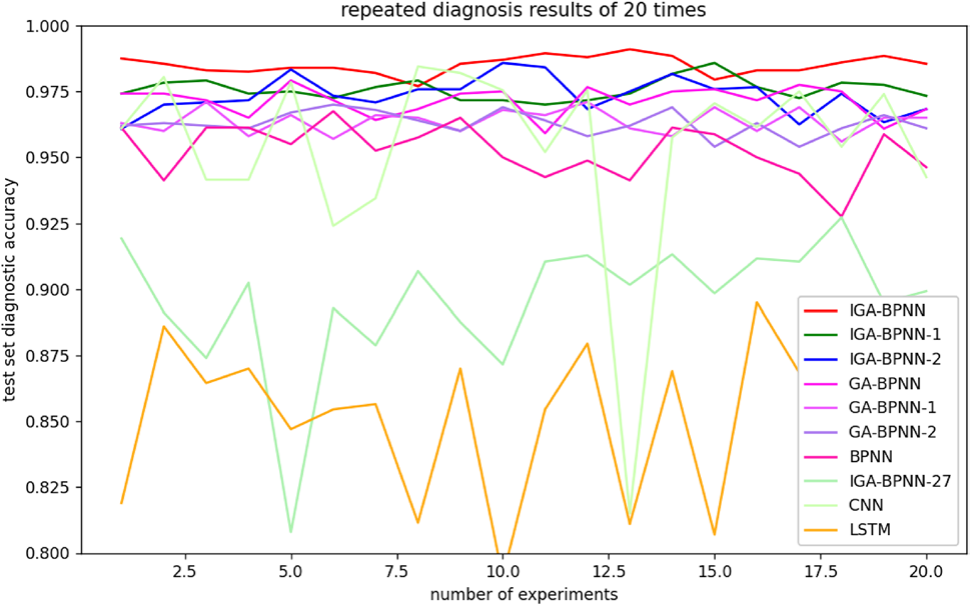
Volatility analysis of eight fault diagnosis algorithms.

Considering the differences in the results of the algorithm under multiple diagnoses, under the same parameter settings and sample proportion allocation, the diagnostic results of the test set sample population are shown in Fig. 12 after 20 repeated calculations. In the figure, it can be observed that the BPNN algorithm exhibits significant fluctuations due to random initialization parameters, with a standard deviation of 0.009791; Other algorithms have optimized the BP neural network, resulting in smoother fluctuations compared to the BPNN algorithm. The standard deviation of IGA-BPNN is 0.003356, while the standard deviations of IGA-BPNN-1, IGA-BPNN-2, GA-BPNN, GA-BPNN-1, and GA-BPNN-2 are 0.003848, 0.006805, 0.005396, 0.004584, and 0.004380, respectively. Although IGA-BPNN-27 uses an improved genetic algorithm to optimize the neural network algorithm, its prediction accuracy is not ideal due to the feature extraction method not following the method proposed in this article, with a standard deviation of 0.024672 and a large fluctuation range. The traditional CNN algorithm and LSTM algorithm, which were not optimized, used the bearing vibration signals extracted in paper^27^ for fault diagnosis. The fluctuation difference of the results was 0.036209 and 0.029874, respectively, with a significantly larger fluctuation range than the aforementioned eight algorithms.It can be seen that the IGA-BPNN algorithm has more stable diagnostic results compared to other algorithms.

### Computational efficiency

**Figure 13:**
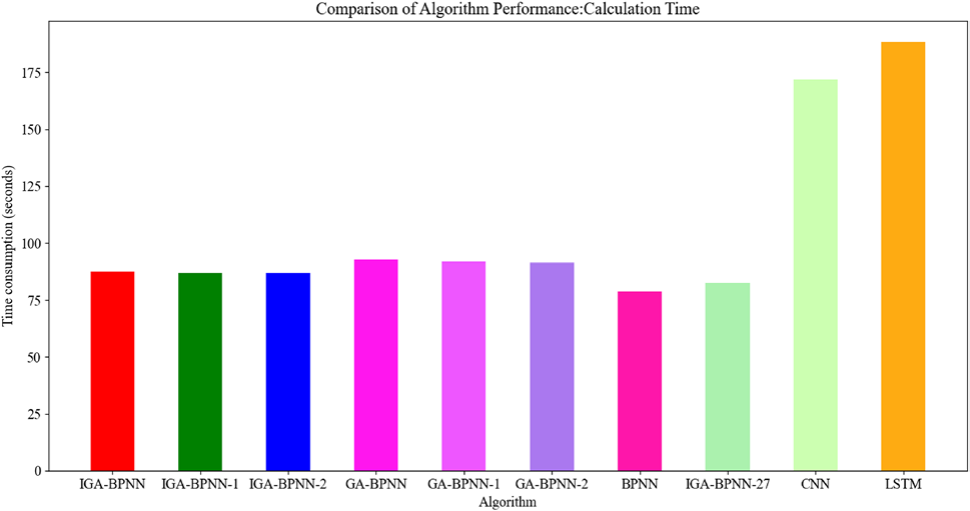
Comparison of computational efficiency of different algorithms.

As shown in Table 9 and Fig. 13, the average computation time required for 10 bearing fault algorithm models, including CNN and LSTM algorithms, in 20 experiments is shown. Among them, IGA-BPNN, IGA-BPNN-1, and IGA-BPNN-2, which both used the improved genetic algorithm proposed in this paper to optimize the traditional BP neural network model, have similar computation time, but all have lower computation time than the GA-BPNN, GA-BPNN-1, and GA-BPNN-2 algorithms that did not improve the genetic algorithm optimized neural network model. The BPNN algorithm lacks genetic selection, crossover, and mutation operations due to the lack of optimization using GA algorithm. Although it takes time to cross, the final diagnostic results of the model are relatively poor. Similarly, IGA-BPNN-27 did not use the 23 features proposed in this article for bearing fault diagnosis. Although its time consumption is relatively low, the diagnostic results of this model are not very ideal. Finally, this article uses the feature signals extracted in paper^27^ to directly input into traditional CNN and LSTM models for fault diagnosis. Due to the complexity of CNN and LSTM results compared to general BPNN neural networks, their time consumption is also the highest among the ten bearing fault diagnosis models.

**Table 9:** Computation time of different fault diagnosis models.

Algorithm	IGA-BPNN	IGA-BPNN-1	IGA-BPNN-2	GA-BPNN	GA-BPNN-1	GA-BPNN-2	BPNN	IGA-BPNN-27	CNN	LSTM
Computation time (s)	87.5	86.9	87.1	92.6	91.8	91.3	78.9	82.7	171.9	188.3

## Conclusions

This article focuses on the optimization of neural network weights and hyperparameters, and proposes a bearing fault diagnosis model based on an improved genetic algorithm IGA optimized BP neural network algorithm from the perspective of evolutionary algorithms. Encode each combination of neural network weights and hyperparameters into a chromosome, iteratively evolve through the IGA algorithm, and ultimately output an optimal combination of network weights and hyperparameters to optimize network performance. The method proposed in this article adopts real number encoding to ensure algorithm accuracy, improve algorithm stability, and through an improved hierarchical proportional selection operator, it not only ensures the optimal preservation strategy but also maintains population diversity, effectively avoiding the problem of genetic algorithm getting stuck in local optima. The convergence of the algorithm is ensured through improved two-point crossover and single point mutation operators, Ultimately, the algorithm has the advantages of fast convergence and avoiding getting stuck in local optima. Finally, the proposed algorithm and seven other fault diagnosis models were trained and evaluated using the bearing fault dataset from Case Western Reserve University, in order to compare and analyze the effectiveness and performance of the IGA-BPNN algorithm proposed in this paper. The experimental results show that the algorithm proposed in this article can fully optimize the combination of neural network weights and hyperparameters, and can obtain the optimal network weights and hyperparameters in a limited time. This provides important reference significance for engineering applications. However, the algorithm proposed in this article still requires more experimental scenarios to verify its effectiveness in different practical application backgrounds.

## Data Availability

The CRWU bearing failure dataset used in this paper is a publicly available dataset that can be downloaded from the following website: https://engineering.case.edu/bearingdatacenter/download-data-file. All data generated or analysed during this study are included in this published article.
